# Changes in C:N:P stoichiometry modify N and P conservation strategies of a desert steppe species *Glycyrrhiza uralensis*

**DOI:** 10.1038/s41598-018-30324-w

**Published:** 2018-08-23

**Authors:** Juying Huang, Pan Wang, Yubin Niu, Hailong Yu, Fei Ma, Guoju Xiao, Xing Xu

**Affiliations:** 1 Institute of Environmental Engineering, Ningxia University, Yinchuan, 750021 China; 2 Ningxia (China-Arab) Key Laboratory of Resource Assessment and Environment Regulation in Arid Region, Yinchuan, 750021 China; 3 College of Resources and Environment, Ningxia University, Yinchuan, 750021 China; 4 Breeding Base for State Key Laboratory of Land Degradation and Ecological Restoration in Northwest China, Ningxia University, Yinchuan, 750021 China

## Abstract

Numerous studies have concluded that carbon (C):nitrogen (N):phosphorus (P) stoichiometry in both soils and plants tends to be decoupled under global change. We consequently hypothesized that plants will adjust nutrient conservation strategies to balance the altered elemental stoichiometry accordingly. To test our hypothesis, we conducted two pot-cultured experiments (with 8-level water and 6-level N addition treatments) using N-fixing species *Glycyrrhiza uralensis* Fisch from a desert steppe in northwestern China. We observed that high water availability lowered total N content and the N:P ratio in soils, further promoting both N and P resorption from senescing leaves of *G. uralensis*. High N addition enhanced soil N availability and the N:P ratio, thereby reducing N resorption, but increasing P resorption of *G. uralensis*. Comparatively, there were also great changes in senescing leaf C:N:P stoichiometry while no clear changes were observed in either green leaf or root C:N:P stoichiometry of *G. uralensis*. As expected, the altered C:N:P stoichiometry may, in turn, modify N and P conservation strategies through their close linkages with N and P uptake in green leaves of *G. uralensis*. This modification may also further exert effects on N and P cycling of the desert steppe.

## Introduction

As consequences of anthropogenic activities, changing precipitation patterns and increasing atmospheric nitrogen (N) deposition have become two serious global environmental problems for terrestrial ecosystems^1^, especially for those limited by precipitation and N. The total amount of global precipitation has reportedly increased in the past 100 years, but drought conditions have also been more frequent in arid and semi-arid regions^2^. Based on synthesized analyses, researchers have observed great differences in the patterns of mean annual precipitation within regions in China in the past several decades^3,4^. Specifically, an increasing precipitation in the west and an aggravating aridity in the east were generally reported across the northwestern regions of China^5,6^. Another serious consequence of anthropogenic activities (i. e. fossil fuel combustion, fertilizer use and intensive animal husbandry), an increased N deposition has accelerated N cycling in ecosystems. Previous studies estimated that the amount of N deposition has reached two to seven times the pre-industrial rates in some developed countries^7^, increasing at a rate of 0.41 kg hm^−2^ yr^−1^ during 1980–2010 in China^8^. Both precipitation and N deposition closely relate to soil nutrient availability and hence regulate nutrient tradeoff between plants and soils^9,10^. Thus, the changes in precipitation and N deposition affect elemental stoichiometry in both plants and soils^11,12^.

Elemental stoichiometry has been suggested as a new approach for studying the interaction between plants and soils, as well as the elemental cycling in plant-soil systems in a changing world^13^. Carbon (C), N, and phosphorus (P) compose the core of elemental cycling, regulating aboveground community composition and also belowground ecological processes. It is generally believed that C:N:P stoichiometry is inherently stable, and thus stabilizes the ecosystem as well^14^. However, many studies have reported that C:N:P stoichiometric balance in both soils and plants tends to be decoupled under intensified global climate (i.e. changing precipitation patterns and increasing N deposition) in recent years^15–18^. C:N:P stoichiometry in both soils and plants could, to a certain extent, indicate nutrient limitation of ecosystems. Specifically, high C:N and low N:P ratios are commonly considered N-limited, while high C:P and N:P ratios mostly represent P limitation. Thus, C:N:P stoichiometry in both soils and plants is said to relate to the efficient use of nutrients in plants^19^. This thereby raises the question of how the decoupling of C:N:P stoichiometric balance affects nutrient conservation strategies of plants, which prolong nutrient resident time in plants and help minimize plant dependence on soil nutrients.

Plant nutrient conservation is considered an important nutrient-use strategy for plants from the most nutrient-poor ecosystems^20^. It determines not only litter decomposition quality, but also soil nutrient availability, and therefore plays a significant role in the nutrient cycling of ecosystems. Both nutrient uptake in green leaves and nutrient resorption from senescing leaves are commonly used parameters to evaluate the ability of nutrient conservation for perennial plants^21^. Generally, low nutrient uptake and/or high nutrient resorption are suggested as useful mechanisms to conserve nutrients for plants in nutrient-poor habitats^22^. However, both nutrient uptake and nutrient resorption by plants are species-specific processes, affected by meteorological factors and vegetation types. Whether nutrient uptake and nutrient resorption can totally explain a plant’s adaptation to nutrient-poor environments is still controversial. Thus far, plant nutrient conservation strategies have been remarkably well-studied in grasslands (i. e., moderate grasslands in northern China), though our knowledge of desert steppe species is still lacking, especially regarding P conservation strategies.

The desert steppe accounts for a large area in the Ningxia Hui Autonomous Region of northwestern China. This ecosystem has been experiencing low precipitation and poor soil N availability, which not only limits plant growth, but also induces high sensitivity to a changing climate. Previous model analyses have revealed that both aridity^6^ and N deposition are continuously increasing^23^ in this region. If the predicted alterations do occur, C:N:P stoichiometric relationships should be changed accordingly. However, both soil and plant C:N:P stoichiometry in desert regions have not yet been sufficiently studied to understand the effects of global change. It also remains unclear how nutrient conservation processes in desert steppe plants respond to altering C:N:P stoichiometry. According to the indication of C:N:P stoichiometry for nutrient limitation and the significance of nutrient conservation strategies in plants, we hypothesized that plants will adjust their nutrient conservation strategies to counteract changes in elemental stoichiometry. To test this hypothesis, we conducted separate water supply (8-level) and N addition (6-level) pot-cultured experiments for desert steppe species *Glycyrrhiza uralensis* from 2011 to 2013. Over three years of treatments, we explored the responses of soil and plant C:N:P stoichiometry and of plant nutrient conservation to changes in water and N regimes. More importantly, we analyzed the associations of soil and plant C:N:P stoichiometry with plant nutrient conservation strategies based on the integrated data from the two experiments. Our results will contribute toward a better understanding of plant adaptation to changing environments in the fragile ecosystems of northern China.

## Results

### Response of the growth of *G. uralensis*

Increasing water supply greatly increased aboveground biomass, root biomass and total biomass of *G. uralensis* with the highest values displayed in W2 treatment (Fig. S1). Increasing N addition also promoted the growth of *G. uralensis* with the highest values shown in N20 treatment (Fig. S2).

### Response of C:N:P stoichiometry in soils

Increasing water supply significantly decreased soil total N content and the N:P ratio, but raised the available N concentration and C:N ratio (Tables 1 and S1). Increasing N addition greatly increased soil total N content, available N concentration and the N:P ratio (Tables 2 and S2). By contrast, neither water supply nor N addition rates consistently influenced soil organic C content, P content or C:P ratio. In the water supply experiment on average, soil organic C content, total N content, total P content, C:N ratio, C:P ratio, and N:P ratio were 7.58 g kg^−1^, 0.76 g kg^−1^, 0.61 g kg^−1^, 10.05, 12.36, and 1.24, respectively, while those in the N addition experiment were 9.54 g kg^−1^, 0.92 g kg^−1^, 0.63 g kg^−1^, 10.61, 15.12, and 1.45, respectively.

**Table 1:** Effects of water supply treatments on soil C:N:P stoichiometry in August, 2013.

Treatments	Organic C (g kg^−1^)	Total N (g kg^−1^)	Total P (g kg^−1^)	C:N ratio	C:P ratio	N:P ratio
W1	7.32 ± 0.34 ab	0.58 ± 0.03 a	0.60 ± 0.03 ac	12.73 ± 0.07 a	12.25 ± 0.36 abc	0.96 ± 0.03 a
W2	7.76 ± 0.38 ab	0.68 ± 0.03 ac	0.62 ± 0.01 ab	11.34 ± 0.13 b	12.53 ± 0.38 abc	1.10 ± 0.02 ab
W3	7.43 ± 0.23 ab	0.81 ± 0.02 b	0.64 ± 0.01 b	9.15 ± 0.15 c	11.57 ± 0.40 ab	1.26 ± 0.04 bc
W4	7.79 ± 0.79 ab	0.81 ± 0.06 b	0.63 ± 0.01 ab	9.53 ± 0.32 cd	12.31 ± 1.19 abc	1.29 ± 0.09 bc
W5	6.96 ± 0.09 a	0.80 ± 0.05 bc	0.63 ± 0.01 ab	8.72 ± 0.53 c	11.06 ± 0.24 a	1.28 ± 0.08 bc
W6	7.38 ± 0.25 ab	0.79 ± 0.03 bc	0.61 ± 0.03 ab	9.37 ± 0.13 cd	12.01 ± 0.11 abc	1.28 ± 0.02 bc
W7	7.56 ± 0.67 ab	0.81 ± 0.05 bc	0.57 ± 0.01 c	9.33 ± 0.30 cd	13.18 ± 1.22 bc	1.41 ± 0.09 c
W8	8.45 ± 0.02 b	0.83 ± 0.05 b	0.61 ± 0.02 ac	10.22 ± 0.65 d	13.96 ± 0.34 c	1.37 ± 0.05 c

W1, W2, W3, W4, W5, W6, W7, and W8 represent water supply rate at 100 mL per 1d, 2d, 3d, 4d, 5d, 6d, 7d, 8d, respectively. Data are presented as means ± SE (*n = *4). Different lowercase letters indicate significant differences between indices within water supply treatments (*P < *0.05). The same lowercase letters indicate insignificant differences (*P* > 0.05).

**Table 2:** Effects of N addition treatments on soil C:N:P stoichiometry in August, 2013.

Treatments	Organic C (g kg^−1^)	Total N (g kg^−1^)	Total P (g kg^−1^)	C:N ratio	C:P ratio	N:P ratio
N0	8.56 ± 0.15 a	0.78 ± 0.05 a	0.62 ± 0.05 a	11.05 ± 0.80 ab	13.94 ± 0.68 ac	1.28 ± 0.13 a
N2.5	10.24 ± 0.49 b	0.83 ± 0.05 a	0.64 ± 0.02 a	12.48 ± 1.26 a	15.91 ± 0.86 ab	1.29 ± 0.07 a
N5	10.55 ± 0.46 b	0.88 ± 0.05 ab	0.62 ± 0.01 a	12.08 ± 0.86 ac	16.90 ± 0.86 b	1.41 ± 0.08 ab
N10	8.43 ± 0.32 a	0.98 ± 0.06 bc	0.61 ± 0.02 a	8.69 ± 0.65 b	13.74 ± 0.43 c	1.60 ± 0.11 b
N20	9.43 ± 0.51 ab	0.99 ± 0.03 bc	0.64 ± 0.02 a	9.57 ± 0.53 b	14.65 ± 0.63 ac	1.53 ± 0.05 ab
N40	10.04 ± 0.33 b	1.02 ± 0.01 c	0.64 ± 0.01 a	9.82 ± 0.45 bc	15.57 ± 0.44 abc	1.59 ± 0.03 b

N0, N2.5, N5, N10, N20, and N40 represent N addition rate at 0, 2.5, 5, 10, 20, and 40 g m^−2^ a^−1^, respectively. Data are presented as means ± SE (*n* = 4). Different lowercase letters indicate significant differences between indices within N addition treatments (*P < *0.05). The same lowercase letters indicate insignificant differences (*P* > 0.05).

### Response of C:N:P stoichiometry in *G. uralensis*

**Figure 1:**
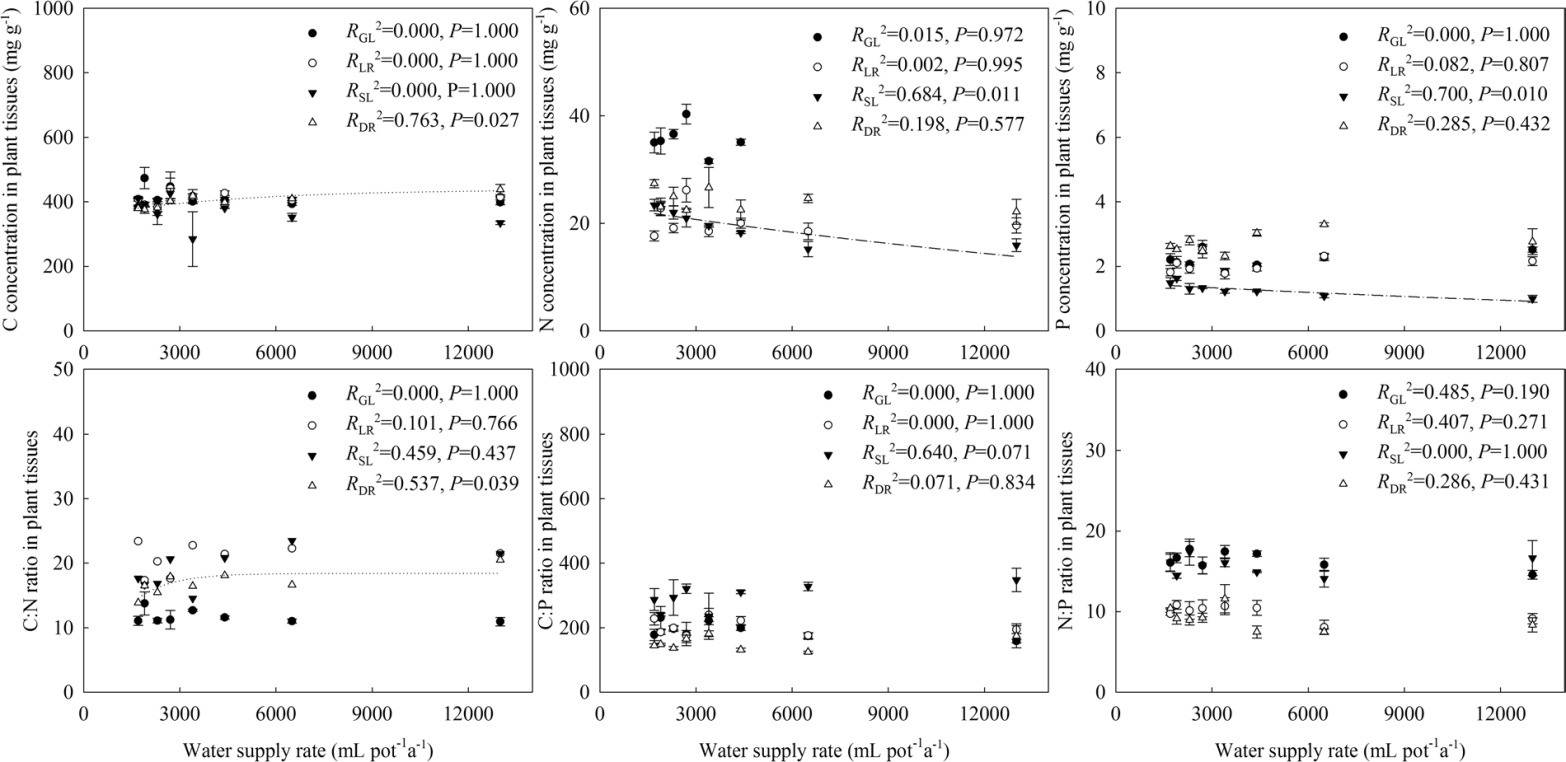
Changes in C:N:P stoichiometry in the leaves and roots of *G. uralensis* along a water supply gradient. Data are presented as means ± SE (*n* = 4). Black circles represent green leaves (GL), white circles represent the roots collected in August (LR), black triangles represent senescing leaves (SL), white triangles represent the roots collected in October (DR).

There were no significant trends in the C:N:P stoichiometry in August samples of *G. uralensis* (green leaves and roots) along the increasing water gradient (Fig. 1). However, across the same water gradient there were exponential increases in C concentration and C:N ratio in October root samples. Exponential decreases were simultaneously observed in N and P concentrations of senescing leaves, indicating that water had increased both NRP and PRP.

**Figure 2:**
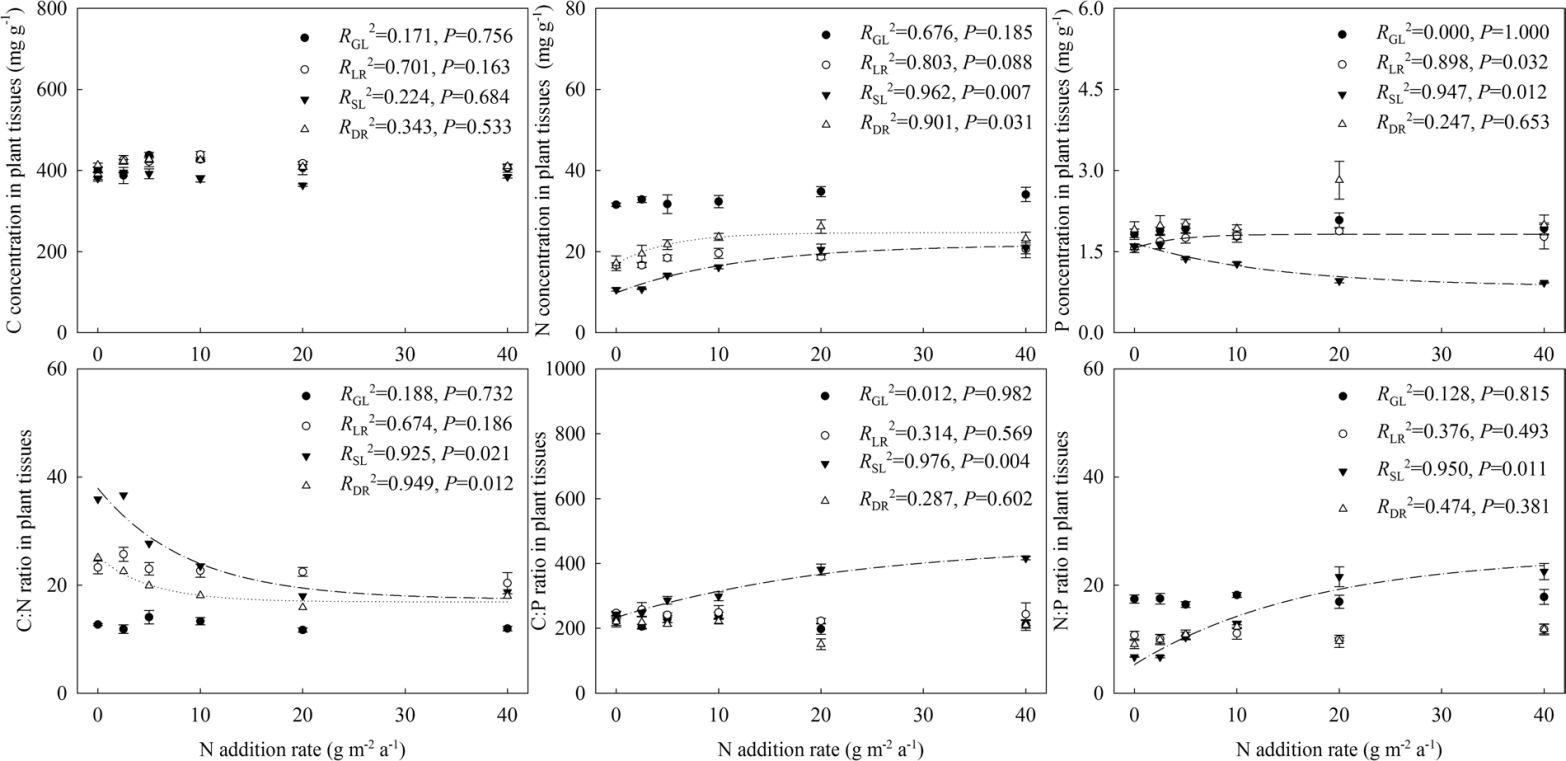
Changes in C:N:P stoichiometry in the leaves and roots of *G. uralensis* along an N addition gradient. Data are presented as means ± SE (*n* = 4). Black circles represent green leaves (GL), white circles represent the roots collected in August (LR), black triangles represent senescing leaves (SL), white triangles represent the roots collected in October (DR).

Along the increasing N addition gradient, C:N:P stoichiometry also had relatively more definitive trends in October samples than in August samples (Fig. 2). For example, N addition increased N concentration, but decreased P concentration in senescing leaves, which resulted in decreasing NRP and increasing PRP.

### Relationship between nutrient conservation and C:N:P stoichiometry

**Figure 3:**
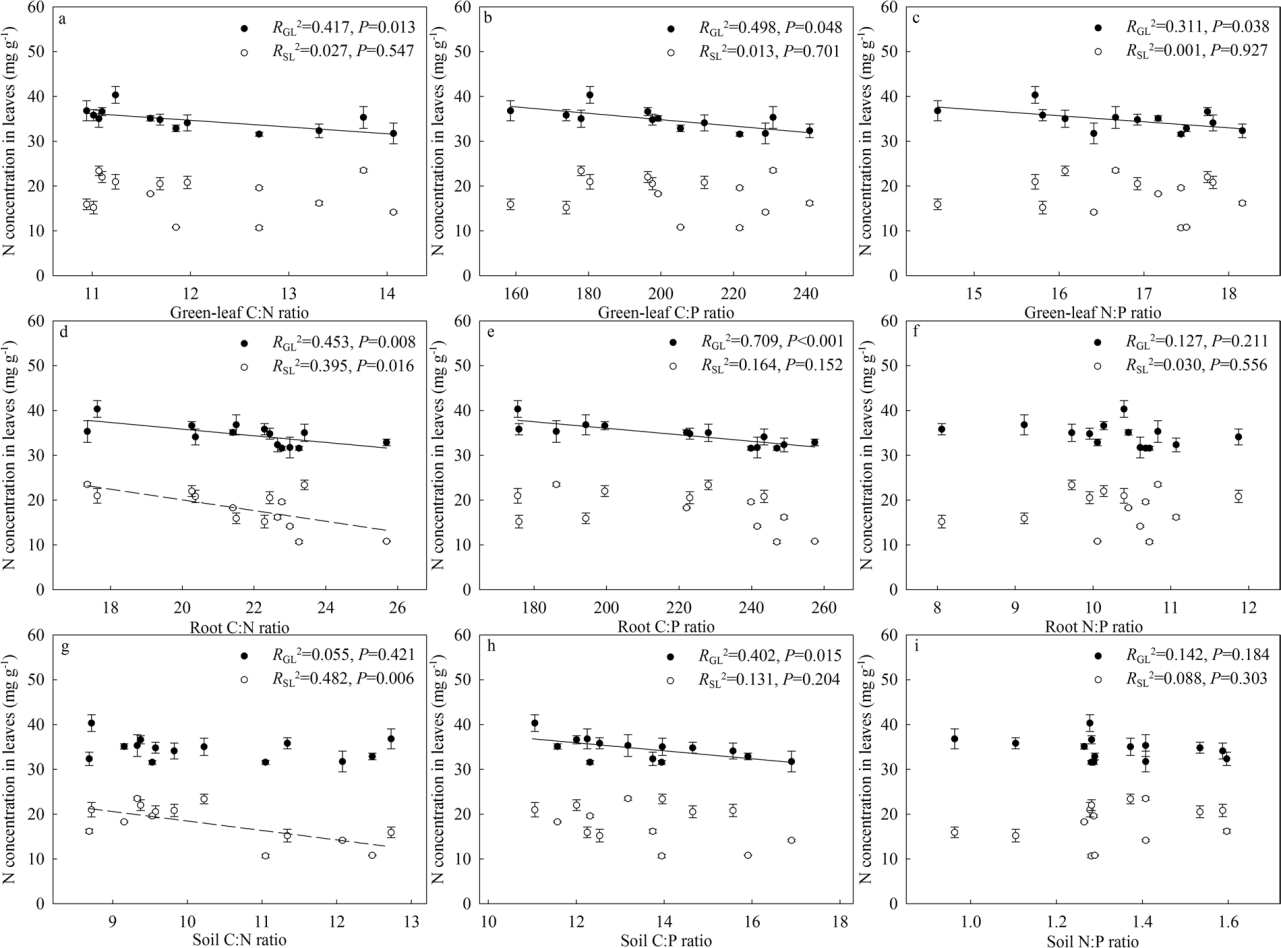
Relationships between N concentration in leaves of *G. uralensis* and C:N:P stoichiometry in August sample (green leaves, roots, and soils). Data are presented as means ± SE (*n* = 4). Black circles represent green leaves (GL), white circles represent the roots collected in August (LR), black triangles represent senescing leaves (SL), white triangles represent the roots collected in October (DR). (**a**–**c**) are for C:N:P stoichiometry in green leaves, (**d**–**f**) are for C:N:P stoichiometry in roots, (**g**–**i**) are for C:N:P stoichiometry in soils.

**Figure 4:**
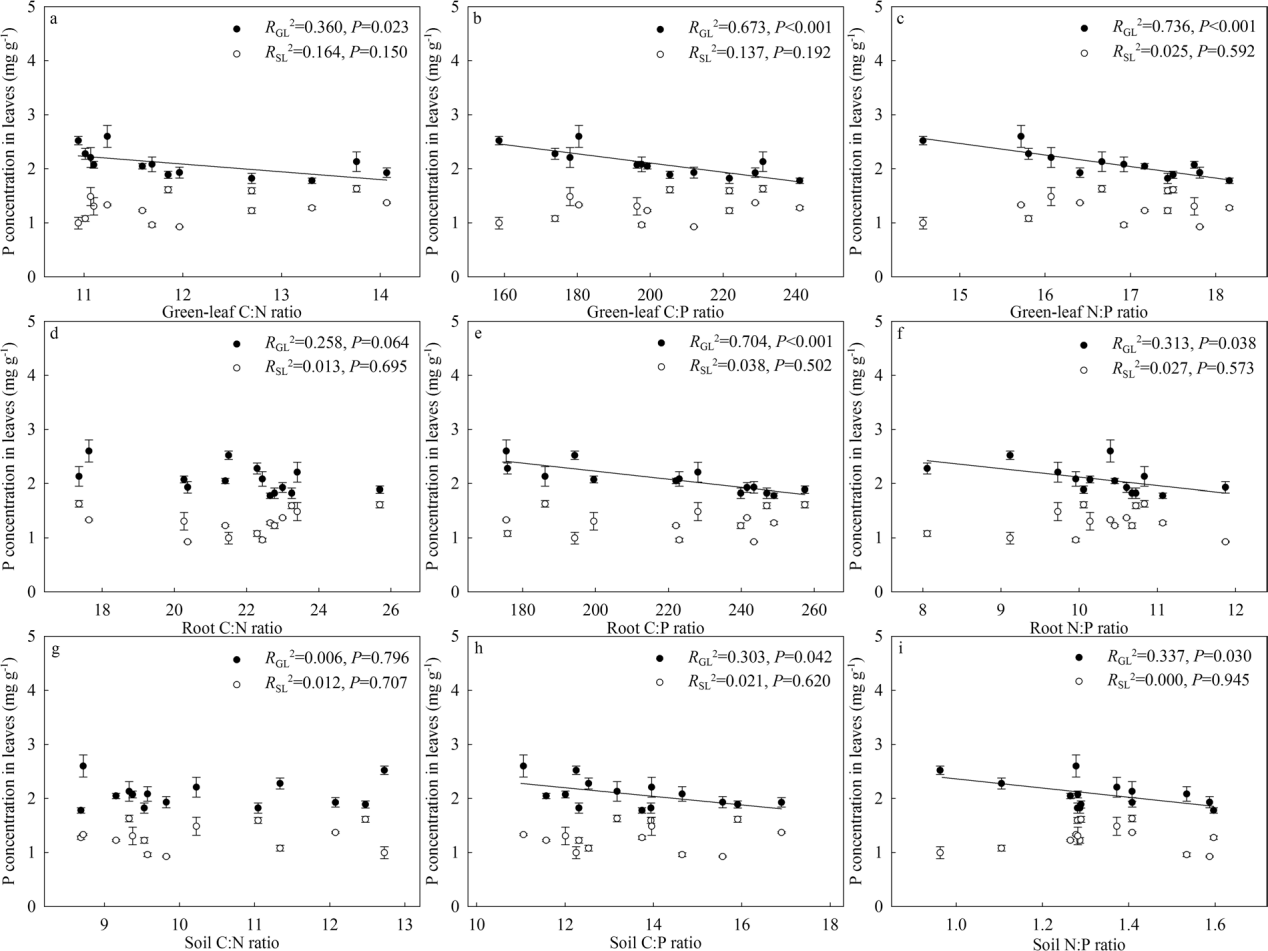
Relationships between P concentration in leaves of *G. uralensis* and C:N:P in August sample (green leaves, roots, and soils). Data are presented as means ± SE (*n* = 4). Black circles represent green leaves (GL), white circles represent the roots collected in August (LR), black triangles represent senescing leaves (SL), white triangles represent the roots collected in October (DR). (**a**–**c**) are for C:N:P stoichiometry in green leaves, (**d**–**f**) are for C:N:P stoichiometry in roots, (**g**–**i**) are for C:N:P stoichiometry in soils.

Both N and P concentrations in green leaves closely correlated with C:N:P stoichiometry in soils, green leaves and roots (Figs 3 and 4). Specifically, N concentration in green leaves was negatively correlated with C:N, C:P and N:P ratios in green leaves, with C:N and C:P ratios in roots, and also with the C:P ratio in soils. P concentration in green leaves decreased with increasing C:N, C:P and N:P ratios in green leaves, with increasing C:P and N:P ratios in roots, and also with increasing C:P and N:P ratios in soils.

Comparatively, both NRP and PRP of *G. uralensis* lacked significant relationships with C:N:P stoichiometry in the three components. Specifically, NRP was only significantly related to the root C:N ratio and the soil C:N ratio, while PRP did not show relate significantly with C:N:P stoichiometry in the three components.

## Discussion

### Changes in C:N:P stoichiometry under different water and N regimes

Both changing precipitation and increasing N deposition can affect soil nutrient availability and hence would closely relate to soil C:N:P stoichiometry. An analysis of a 3500 km transect through a temperate climate in northern China revealed that soils from more arid sites were associated with a lower soil organic C content, N content, P content, C:P ratio and N:P ratio, but a higher C:N ratio, which suggests that predicted precipitation changes could lead to soil C:N:P imbalance^24^. The same trends in N content and N:P ratio were also reported across grassland soils in the central great plains of North America^25^ and the forest soils in China^26^. Generally, short-term N addition had no consistent effects on C and P content in soils, but led to an increase in soil N availability^27^, which in turn resulted in an increased N:P ratio and a decreased C:N ratio in soils^12,28^. In our work, we also found that N addition increased soil N availability and the N:P ratio (Tables 2 and S2). In contrast, an increasing water supply enhanced soil available N, but had little effect on soil total N content. However, an excess of water (watering frequencies were 100 mL per 1d and 2d) led to sharp decreases in soil total N availability (Tables 1 and S1), possibly because the soil in the study was sandy, which is characterized by a poor ability to conserve water and nutrients. This much water likely caused substantial soil N loss via leaching^29^, consequently causing a great increase in the C:N ratio, as well as a decrease in the N:P ratio (Table 1).

Previous studies have pointed out that plant N concentration is more sensitive than C and P concentrations to changing precipitation^30^ and N regimes^11,28^, which results in clearer responses in the N:P ratio than the C:N and C:P ratios. However, the patterns of plant C:N:P stoichiometry are species-specific and scale-dependent. An analysis based on a global dataset showed that drought stress increased the plant N:P ratio^18^, whereas the study on *Phragmites australis* across northeastern China reported a decreasing N:P ratio and increasing C:N and C:P ratios along a decreasing precipitation gradient^31^. Comparatively, short-term N addition can generally promote plant N uptake^17^. However, a long-term or high-pulse input of N will cause an increase in plant P demand^32^. Similarly, increasing N deposition also frequently widens the N:P ratio and narrows the C:N ratio in leaves and roots^12,17,18,33^. In the present study, we observed that both water supply and N addition had unclear influences on the C:N:P stoichiometry in *G*. *uralensis*, except in the senescing leaves (Figs 1 and 2), which is inconsistent with the significant changes observed in some non N-fixing species subjected to altered precipitation patterns^31,34^ and artificial N addition^33,35^. This may be because the roots of leguminous plants can build symbiotic relationships with nodule bacteria, which are of great benefit for fixing free N from the air. Consequently, the self-adjusting N economy means that leguminous plants are less affected by three years of change in water supply and N addition. This relatively stable C:N:P stoichiometry in *G. uralensis* might help maintain a high elemental internal balance and a low sensitivity to changing environments.

### Changes in nutrient conservation strategies in leaves of *G. uralensis* under different water and N regimes

A commonly held idea suggests that a low nutrient concentration in green leaves and/or a high nutrient resorption from senescing leaves are effective approaches to conserve nutrients for species from nutrient-limited habitats, often reflecting the adaptation of plants to regional climatic factors^36^. However, the trends in these two indicators in response to precipitation always change with species and depend on the study scale. For example, the results from a large-scale dataset showed that nutrient uptake decreases with increasing precipitation^11,37,38^, whereas other studies found that increased precipitation enhances the transformation of nutrients in soils and thus promoted the absorption of nutrients by plants^31,39^. Some studies observed that N resorption decreased and that P resorption increased along increasing precipitation gradients^40,41^, whereas others reported that neither NRP nor PRP was correlated with precipitation across three types of forests^42^. In the present study, we found that both water supply and N addition had unclear effects on N and P uptake in *G. uralensis*, which is inconsistent with the previously reported responses of a grass species, *Stipa purpurea*^43^ and two shrubs^44^. In contrast, excessive water led to an increase in both N and P resorption of *G. uralensis*, which is contrary to the responses of species from the tropical dry forest^45^ and from the temperate grassland^46^. N addition decreased N resorption, but increased P resorption of *G. uralensis*, which is similar to the results of grass species *Seriphidium rhodanthum*^47^. These findings likely resulted from water-induced losses in N and P (Tables 1 and S1) and N-induced increases in P limitations (Table 2). Our results indicate that *G. uralensis* could positively adjust its nutrient resorption approach, but not its nutrient uptake approach, to alleviate nutrient deficiency induced by an excessive water supply and high N addition.

### Linkages between C:N:P stoichiometry and nutrient conservation strategies

C:N:P stoichiometry in both soils and plants may indicate nutrient limitation types of plant growth^48,49^. Thus, the C:N:P stoichiometry of soils and plants are supposed to closely correlate with N and P strategies of plants. Usually, both high C:N ratio and low N:P ratio represent N limitation, while high C:P and N:P ratios imply P limitation. According to the concept of nutrient conservation strategy, high N conservation abilities of plants are supposed to occur at a high C:N ratio and a low N:P ratio in soils and plants. On the other hand, high P conservation abilities of plants may occur at high C:P and N:P ratios. In the present study, we analyzed the associations of *G. uralensis* with integrated C:N:P stoichiometry in August samples (soils, green leaves, and roots) from the two experiments in both nutrient uptake in green leaves and nutrient resorption in senescing leaves (Figs 3 and 4). We observed that N uptake was generally negatively correlated with the C:N and C:P ratios in soils, green leaves and roots, while P uptake was negatively correlated with the C:P and N:P ratios in the same three components. Contrary to our expectations, both NRP and PRP had unclear relationships with the C:N:P stoichiometry in all three components. Our results might indicate that shifts in C:N:P stoichiometry, which are induced by changing water supply and N addition regimes, possibly modify nutrient conservation strategies of *G. uralensis* through their close linkages with nutrient uptake. The altered nutrient uptake abilities may, in turn, reflect the self-adjusting adaptation of *G. uralensis* to the changing C:N:P stoichiometric balance.

Taken together, our results showed that three years of low water supply and N addition had little effects on elemental stoichiometric balance, whereas high amounts of both water supply and N addition greatly altered the C:N:P stoichiometry in soils and senescing leaves of *G. uralensis*, which indicates that excessive water supply and high N addition lead to an imbalance in the C:N:P stoichiometry in soils and in *G. uralensis*. As expected, the altered elemental stoichiometry might modify N and P conservation strategies of *G. uralensis* through their influence on N and P uptake approaches, thereby leading to important feedbacks on N and P cycling in the desert steppe in northwestern China. Our results could provide a scientific guide for the adaptive management of the desert steppe in a changing global climate. In light of the N-fixing characteristics of the studied species, long-term simulated field experiments that focus on the comparisons among different functional groups are urgently needed to better understand the effects of climate change on the stability of fragile ecosystems.

## Material and Methods

### Study site and species selection

The pot-cultured experiments were conducted in Yanchi Desert Steppe Station (37.1°−38.2°N, 106.5°′−107.7°E, 1380–1600 m a.s.l.) in the Ningxia Hui Autonomous Region, which is located in the eastern part of northwestern China. This area has a typical continental climate in the moderate temperate zone and lies on the southwest edge of the Mu Us Sandy Land. The mean annual precipitation is 289 mm, with over 70% falling during the growing season (May to September). The mean annual evaporation is 2132 mm. The mean temperatures are −8.9 °C in January and 22.5 °C in July. The major soil type is Aridisol (FAO classification). The main vegetation type is desert steppe, which is mainly composed of grasses and shrubs, such as *Lespedeza potaninii*, *Cynanchum komarovii*, *Pennisetum centrasiaticum*, *Cleistogenes squarrosa*, *Agropyron cristatum*, *Stipa capillata*, *Glycyrrhiza uralensis*, *Oxytropis aciphylla*, *G. uralensis* and *Caragana korshinskii*.

*G. uralensis* was selected for its ability to fixate N. *G. uralensis* is used in the desert steppe of Ningxia to protect against the wind, to fixate sand and as a traditional Chinese Medicine. In recent years, the degradation of *G. uralensis* vegetation has been aggravated by intensified climate change and anthropogenic disturbance (i.e., aridity and grazing) in this region. Wild *G. uralensis* resources have also been overused due to their important economic values. The artificial cultivation of *G. uralensis* has been widely promoted since the implementation of a national policy prohibiting over-digging. Nowadays, Yanchi, Hongsibao, Pingluo, Zhongning and other *G. uralensis* plantations have been introduced in Ningxia.

### Experimental design

The two pot-cultured experiments were carried out during 2011–2013. In late March 2011, 224 free-draining polyvinyl chloride pots (16 cm in diameter, 50 cm in height) were vertically buried in soils, leaving 50 mm aboveground surface. 0–50 cm depth soils were collected from *G. uralensis* plantations at the Yanchi Desert Steppe Station and were sieved to remove small stones and plant litter. Each pot was filled with about 11.0 kg of soils according to soil bulk density. Before transplantation, soil organic C, total N, and total P contents were analyzed, their values measured at 11.64 g kg^−1^, 0.63 g kg^−1^ and 0.65 g kg^−1^, respectively. Seedlings with a uniform morphology were also collected from *G. uralensis* plantations, specifically 10–20 cm in height, 0.4–0.5 cm in stem diameter, and with 5–6 leaves. In each pot, two seedlings were planted together. After two weeks of pot-transplantation, only the more vigorous individual was kept.

Both water supply and N addition treatments began the fifth week after transplanting and were conducted from May to August in each year. To test whether and to what extent water supply and N addition could affect both C:N:P stoichiometry and nutrient conservation, eight treatments and six treatments were designed for water supply and N addition experiments, respectively. Each treatment had eight replicates in both experiments. The eight treatments in the water supply experiment were 100 mL of water per 1d 2d, 3d, 4d, 5d, 6d, 7d, and 8d, respectively. The total water supply and equivalent precipitation for each treatment were presented in Table 3.

**Table 3:** The total water supply and equivalent precipitation for each treatment.

Treatments	W1	W2	W3	W4	W5	W6	W7	W8
Total water supply (mL pot^−1^ a^−1^)	13000	6500	4400	3400	2700	2300	1900	1700
The equivalent precipitation (mm a^−1^)	1368.6	684.3	463.2	356.0	284.3	242.1	200.0	179.0

W1, W2, W3, W4, W5, W6, W7, and W8 represent water supply rate at 100 mL per 1d, 2d, 3d, 4d, 5d, 6d, 7d, 8d, respectively.

The six treatments in the N addition experiment were 0, 2.5, 5, 10, 20, and 40 g N m^−2^ a^−1^. N fertilizer was added as NH_4_NO_3,_ which has 34% N composition. To increase N use and avoid N poisoning, NH_4_NO_3_ was dissolved first in water, and then applied 2–4 times per week. A rain shelter covered with blue polyvinyl chloride film, where the light transmittance is more than 95%, was used to exclude natural precipitation in the water supply experiment. The rain shelter was kept ventilated while it was no rain. All pots were coverless and received the same precipitation in the N addition experiment. During the experimental period, weeds were pulled from pots weekly for both experiments.

### Collection of samples

Thirty fully expanded green leaves free from disease and insect damage and the rest of the aboveground organs of *G. uralensis* were collected from each pot in late August 2013, while thirty attached senescing leaves (yellow and ready to drop) were collected in early November 2013. The root samples of *G. uralensis* were simultaneously harvested in the two seasons of 2013. 0–10 cm soil samples were collected using a soil-drilling (30 mm in diameter) in late August 2013. During each harvest, four replicates were randomly selected from each treatment. All samples were stored in an insulated can and immediately taken to the laboratory for further analysis.

### Laboratory methods and calculations

Both August and October samples of *G. uralensis* were dried in an oven (65 °C for 48 h), then the dry weight of August samples was measured to calculate aboveground biomass per pot. Root samples were rinsed with distilled water and then dried at 75 °C for 48 h to measure their dry weight per pot. C, N, and P concentrations were analyzed after the samples were finely ground in a Wiley mill and passed through a 40-mesh screen. C concentration was measured with the K_2_MnO_4_ volume method. N concentration was analyzed by the Kjeldahl acid–digestion method, using an Alpkem AutoAnalyzer (Kjektec System 2300 Distilling Unit, Sweden). P concentration was determined colorimetrically at 700 nm after reacting with a molybdenum antimony solution. The nutrient concentration of senescing leaves was used to evaluate nutrient resorption proficiency (RP) because high nutrient concentration in the senescing leaves of a plant means that a plant has a low RP. In the present study, N resorption proficiency and P resorption proficiency are abbreviated as NRP and PRP, respectively.

Soil samples were separated into two parts, where one part was for measuring organic C (K_2_MnO_4_ volume method), total N (Kjeldahl method) and total P contents (HCLO_4_-H_2_SO_4_ method) after air drying. The second part was kept fresh for measuring immediately available N (Alkali solution diffusion) and available P concentrations (0.5 mol L^–1^ NaHCO_3_ method)^50^.

### Statistical analysis

Statistical analyses were performed in SPSS version 13.0 (SPSS Inc., Chicago, IL, USA). The K-S test was used to confirm normality. A one-way ANOVA with least significant difference (LSD) post-hoc tests was used to analyze the effects of changing water and N regimes on the biomass of *G. uralensis* and soil C:N:P stoichiometry. Exponential regressions were used to describe the changes in C:N:P stoichiometry in leaves and roots along a water supply rate gradient and an N addition rate gradient, respectively. Linear regressions were used to analyze the relationships between leaf nutrient concentration and C:N:P stoichiometry in August samples (green leaves, roots, and soils).

## Electronic supplementary material

Dataset 1
