# Supplementary material for: Changes in C:N:P stoichiometry modify N and P conservation strategies of a desert steppe species Glycyrrhiza uralensis
Source: Sci Rep. 2018 Aug 23;8:12668. doi: 10.1038/s41598-018-30324-w (PMC6107674; doi:10.1038/s41598-018-30324-w)
Supplement: Supplementary file 1 — Dataset 1 [file 41598_2018_30324_MOESM1_ESM.pdf]

SREP-18-07036A

Changes in C:N:P stoichiometry modify N and P conservation strategies of a desert steppe species *Glycyrrhiza uralensis*

Juying Huang[1, 2], Pan Wang[3], Yubin Niu[3], Hailong Yu[2, 3], Fei Ma[1, 2], Guoju Xiao[1, 2], Xing Xu[4*]

[1]Institute of Environmental Engineering, Ningxia University, Yinchuan 750021, China

[2]Ningxia (China-Arab) Key Laboratory of Resource Assessment and Environment Regulation in Arid Region, Yinchuan 750021, China

[3]College of Resources and Environment, Ningxia University, Yinchuan 750021, China

[4]Breeding Base for State Key laboratory of Land Degradation and Ecological Restoration in Northwest China, Yinchuan 750021, China

Table S1 Effects of water supply treatments on soil available N and P concentrations in August, 2013.

Indices	W1	W2	W3	W4	W5	W6	W7	W8
Available N (mg kg^{-1})	7.54±0.95 a	9.07±0.68 a	13.47±1.27 b	13.01±1.00 b	10.15±0.08 ab	10.07±2.09 ab	6.72±1.02 a	6.95 ± 1.07 a
Available P (mg kg^{-1})	6.09±0.65 a	9.01±1.85 a	15.25±4.08 b	8.48±1.10 a	7.61±0.78 a	9.45 ± 1.92 ab	7.35±0.44 a	10.99±2.88 ab

W1, W2, W3, W4, W5, W6, W7, and W8 represent water supply rate at 100 mL per 1d, 2d, 3d, 4d, 5d, 6d, 7d, 8d, respectively. Data are presented as means ±SE (n = 4). Different lowercase letters indicate significant differences between the same indices within water supply treatments ($P < 0.05$). The same lowercase letters indicate insignificant differences ($P > 0.05$).

Table S2 Effects of N addition treatments on soil available N and P concentrations in August, 2013.

Indices	N0	N2.5	N5	N10	N20	N40
Available N (mg kg^{-1})	10.15±0.08 a	12.56±2.17 a	15.18±1.80 a	39.25±1.18 b	38.78±4.53 b	39.48±2.31 b
Available P (mg kg^{-1})	9.35±0.44 a	10.59±0.62 a	12.53±2.17 a	9.93±0.98 a	11.44±1.38 a	13.32±1.84 a

N0, N2.5, N5, N10, N20, and N40 represent N addition rate at 0, 2.5, 5, 10, 20, and 40 g m^{-2} a^{-1}, respectively. Data are presented as means ±SE (n = 4). Different lowercase letters indicate significant differences between the same indices within N addition treatments (P < 0.05). The same lowercase letters indicate insignificant differences (P > 0.05).

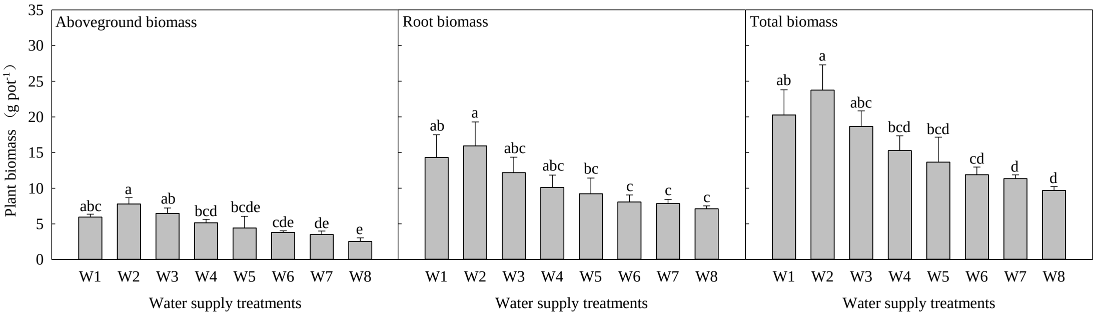

Figure S1 Effects of water supply treatments on aboveground biomass, root biomass, and total biomass of *G. uralensis*. W1, W2, W3, W4, W5, W6, W7, and W8 represent water supply rate at 100 mL per 1d, 2d, 3d, 4d, 5d, 6d, 7d, 8d, respectively. Data are presented as means ± SE (n = 4). Different lowercase letters indicate significant differences between the same indices within water supply treatments ($P < 0.05$). The same lowercase letters indicate insignificant differences ($P > 0.05$).

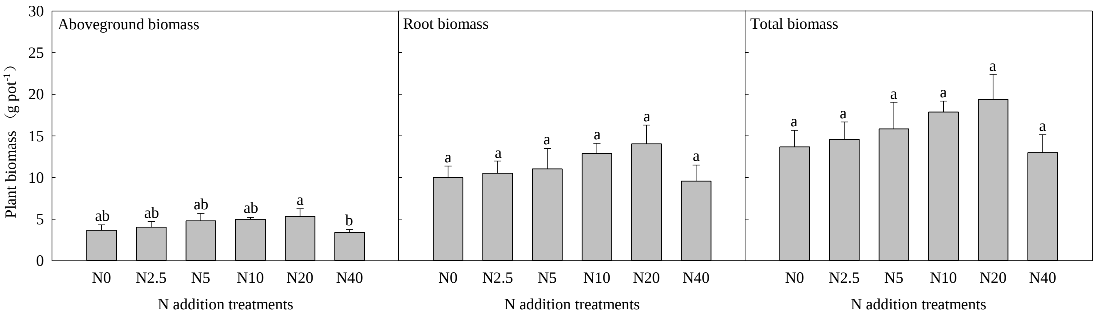

Figure S2 Effects of N addition treatments on aboveground biomass, root biomass, and total biomass of *G. uralensis*. N0, N2.5, N5, N10, N20, and N40 represent N addition rate at 0, 2.5, 5, 10, 20, and 40 g m^{-2} a^{-1}, respectively. Data are presented as means ± SE (n = 4). Different lowercase letters indicate significant differences between the same indices within N addition treatments ($P < 0.05$). The same lowercase letters indicate insignificant differences ($P > 0.05$).
